# MECHANISMS OF CHANGE IN COGNITIVE FUNCTION DOMAINS IN OLDER ADULTS

**DOI:** 10.1093/geroni/igad104.0657

**Published:** 2023-12-21

**Authors:** Zheng Zhu, Jessica Zwerling, Xiang Qi, Yaolin Pei, Yaguang Zheng, Bei Wu

**Affiliations:** New York University, New York City, New York, United States; Albert Einstein College of Medicine, Bronx, New York, United States; New York University, New York City, New York, United States; New York University, New York City, New York, United States; New York University, New York City, New York, United States; New York University, New York City, New York, United States

## Abstract

It is crucial to gain a better understanding of these relationships in order to have a more comprehensive understanding of cognitive abilities over time and how interventions can target multiple domains to improve overall cognitive function. This study aimed to investigate the mechanism of changes in cognitive function domains in phenotypic networks. This study used data from the Aging, Demographics, and Memory Study (ADAMS) in Wave A and Wave B. We assessed 12 cognitive function domains. Latent profile transition analysis (LPTA) and cross-lagged panel network model were employed to the dynamic interactions of the 12 cognitive function domains over time in both the deterioration and improvement groups. A total of 252 participants were included in the final analysis. LPTA identified five subgroups and categorized all samples into three main categories: improvement group (n=61), deterioration group (n=54), and no change group (n=137). “D9: psychomotor processing” showed the largest value of out-strength in the deterioration group (r=0.941) and improvement group (r=0.969). The strongest direct positive effect in the deterioration group was “C9: psychomotor processing” -> “C8: attention” (β=0.39). In the improvement group, the strongest direct positive effect was “C9=psychomotor processing” -> “C7=visual memory” (β=0.69). Psychomotor processing can affect other cognitive domains, and it plays a crucial role in changes of cognitive function. The paths of psychomotor processing to attention and visual memory were found to be major factors in cognitive deterioration and improvement. Targeting psychomotor processing may lead to the development of more effective and precise interventions.

